# Posterior Interosseous Nerve Palsy Caused by Parosteal Lipoma: A Case Report

**DOI:** 10.1155/2010/785202

**Published:** 2010-08-08

**Authors:** Hatem Salama, Pradeep Kumar, Salah Bastawrous

**Affiliations:** ^1^Royal Derby Hospital, Uttoxeter Road, Derby DE22 3NE, UK; ^2^Glan Clwyd Hospital, Rhyl LL18 5UJ, UK

## Abstract

An 83-year-old woman presented with weakness in her right-hand and wrist extensors and swelling in the proximal part of the right forearm. Electromyography (EMG) confirmed involvement of posterior interosseous nerve at the level of proximal forearm. MR imaging demonstrated the characteristics of lipoma which extended on the anterolateral aspect of the right forearm and at the level of the radius neck. The lesion was parosteal lipoma causing compression and paralysis of the posterior interosseous nerve without sensory deficit. In this paper, posterior interosseous nerve palsy due to compression of a parosteal lipoma recovered after excision of the lipoma followed by intensive rehabilitation for six month. Surgical excision should be performed to ensure optimal recovery from the nerve paralysis.

## 1. Introduction

Lipomas are common benign soft-tissue tumours which are usually asymptomatic [1]. Sometimes, when they exist in deeper planes, they might cause compression of adjacent structures including nerves [2]. Posterior interosseous nerve palsy without any history of previous trauma is an uncommon condition but does occur due to spontaneous entrapment [3]. However, a parosteal lipoma, occurring adjacent to the proximal end of radius, may cause paralysis of the posterior interosseous nerve because of the specific anatomical relationship between different structures in that area. The posterior interosseous nerve passes between the superficial and deep layers of the supinator muscle, and hence, the nerve is vulnerable because of possible entrapment against the proximal edge of the muscle (arcade of Frohse) [4].

## 2. Case Presentation

An 83-year-old woman presented with acute and progressive weakness of the right-hand extensors with painless swelling in the proximal part of the right forearm. Interestingly, she noticed that weakness after a fall on the outstretched left hand leading to fracture of left distal radius. She was treated conservatively in a cast. Six weeks later, and during routine follow-up, she mentioned that she noticed her right-hand weakness only when she started to use it excessively. She denied any injury to her right-hand or elbow. Examination revealed a swelling 3 × 3 cm in the antero-lateral aspect of the right forearm in the region of supinator muscle. The swelling was soft in consistency and decreased in size on flexion with supination of the forearm indicating that the lump was deep to the brachioradialis muscle. Extension of the metacarpophalangeal joints of all fingers in her right-hand was weakened. Also, there was slight weakness in the wrist joint extension. There was no sensory deficit in any of the dermatomes of the hand or the forearm. 

**Figure 1:**
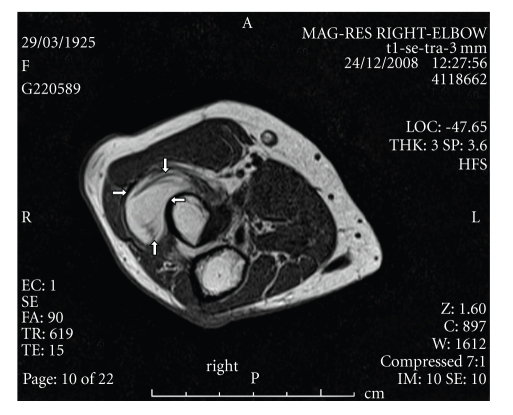
T1-weighted axial MR images reveal a high-intensity lesion around the radius, white arrows.

**Figure 2:**
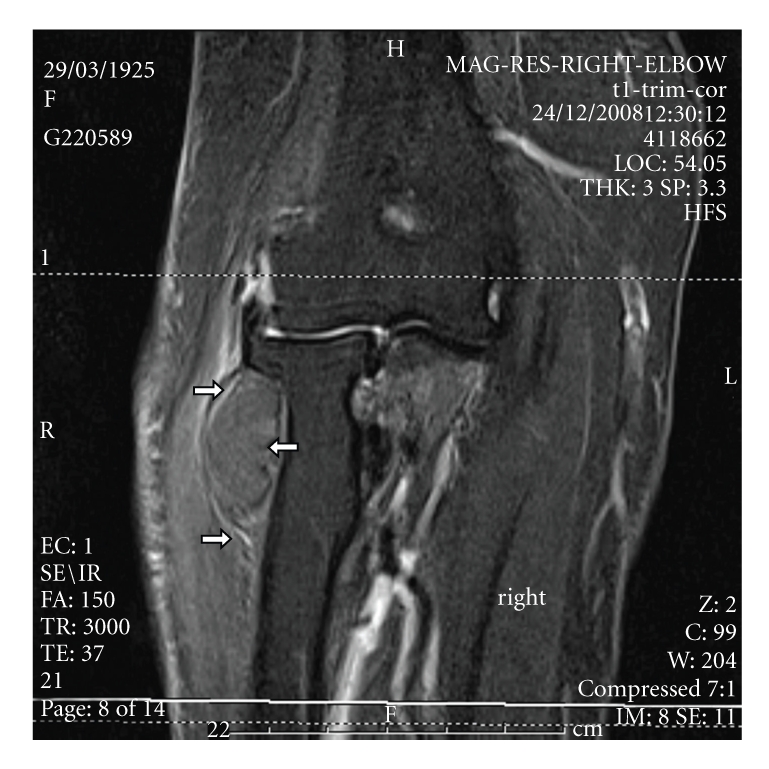
T1-weighted coronal MR image reveals the displacement exerted on surrounding structures by the lesion around the radius, white arrows.

Radiographs of the right elbow showed soft-tissue density closely related to the proximal radius with normal bone appearance. Electromyography of the posterior interosseous nerve and muscles demonstrated active denervation, but nevertheless there were 1 or 2 recruited motor units confirming continuity of these motor fibres. Magnetic resonance imaging (MRI) of the right forearm revealed a multilobulated mass with hypointensity signal on a T1-weighted sequence which is pathognomonic of a lipoma. The precise anatomical location was in the lateral and anterior aspects of the right radius neck and extending distally for about 3 cm as shown in Figures 1 and 2. The lesion was explored through anterior approach under general anaesthesia. The radial nerve and its two branches were identified. PIN was traced distally to the level of the proximal margin of volar supinator muscle (arcade of Frohse). There was focal constriction of the nerve at the level of the proximal edge of the supinator muscle. The nerve was slightly atrophied distal to this constriction. The attachment of the supinator muscle was dissected from the radius. The lesion was deep to supinator and was encapsulated and firmly attached to the proximal radius. The lesion then was excised with stripping of the attached periosteum. Histology confirmed the diagnosis of lipoma which was totally encapsulated except at the site of its attachment to bone. 

Postoperatively, with intensive physiotherapy, she noticed improvement in hand and wrist extension. The final check, 6 months later, confirmed that elbow, wrist, and hand function were similar in both sides.

## 3. Discussion and Conclusion

Compression or entrapment of the posterior interosseous nerve may occur at various anatomical sites in the forearm and for various reasons. At the level of proximal forearm, it can be caused by lipomas, fibromas, arteriovenous malformation, bursa, ganglion, or as a result of synovial cysts in osteoarthrosis or rheumatoid arthritis. In addition, focal constriction of the nerve may occur without trauma or external compression [5–8]. 

Involvement of PIN by lipomas is unusual and is characterized by a gradual onset of symptoms without sensory loss [1, 9]. The superficial sensory radial (SSR) has a more superficial and medial course than the posterior interosseous nerve (PIN). In this case report, the acute presentation of weakness may be because the patient was no longer able to use her left hand after fracture and cast application, and that made weakness in the right side more evident. However, Bugnicourt et al. reported one patient with acute symptoms of posterior interosseous nerve compression without any history of trauma [10].

Parosteal lipoma often has distinctive features that allow diagnosis on radiographs. MR imaging is the procedure of choice following radiography because of its multiplan imaging capabilities and improved ability to show muscle atrophy caused by the compression of adjacent nerves. These factors have significant implication for improving preoperative assessment and for helping to guide surgical treatment. The classical finding of parosteal lipoma on plain radiographs is a radiolucent lesion in the soft tissue closely applied to the subjacent bone with or without some osseous changes [11]. As in this case, MRI imaging gave a precise preoperative diagnosis delineating the relationship of the lesion with the surrounding structures. 

Surgical excision of parosteal lipoma of the proximal radius has been recommended in the literature to prevent posterior interosseous nerve compression and to increase the likelihood of functional recovery as the recovery of the neurological deficit relates to the duration of symptoms. The prognosis after excision of parosteal lipoma is excellent, with very low recurrence rates [12, 13].

In conclusion, parosteal lipoma occurring in the proximal radius may readily cause paralysis of the posterior interosseous nerve. Surgical excision might be required to prevent compression of the posterior interosseous nerve and facilitate neurological recovery.
